# TRAIL/TRAIL Receptor System and Susceptibility to Multiple Sclerosis

**DOI:** 10.1371/journal.pone.0021766

**Published:** 2011-07-21

**Authors:** Carlos López-Gómez, Óscar Fernández, Juan Antonio García-León, María Jesús Pinto-Medel, Begoña Oliver-Martos, Jesús Ortega-Pinazo, Margarita Suardíaz, Lucía García-Trujillo, Cristina Guijarro-Castro, Julián Benito-León, Isidro Prat, Jezabel Varadé, Roberto Álvarez-Lafuente, Elena Urcelay, Laura Leyva

**Affiliations:** 1 Research Laboratory, Clinical Neurosciences Institute, Hospital Regional Universitario Carlos Haya and Fundación IMABIS, Málaga, Spain; 2 Department of Neurology, Clinical Neurosciences Institute, Hospital Regional Universitario Carlos Haya and Fundación IMABIS, Málaga, Spain; 3 Department of Neurology, University Hospital 12 de Octubre, Madrid, Spain; 4 Centro de Investigación Biomédica en Red sobre Enfermedades Neurodegenerativas, Madrid, Spain; 5 Department of Medicine, Complutense University, Madrid, Spain; 6 Transfusion Center Blood Bank, Málaga, Spain; 7 Department of Immunology, Hospital Clínico San Carlos, Instituto de Investigación Sanitaria San Carlos (IdISSC), Madrid, Spain; 8 Department of Neurology, Hospital Clínico San Carlos, Instituto de Investigación Sanitaria San Carlos (IdISSC), Madrid, Spain; Julius-Maximilians-Universität Würzburg, Germany

## Abstract

The TNF-related apoptosis inducing ligand (TRAIL)/TRAIL receptor system participates in crucial steps in immune cell activation or differentiation. It is able to inhibit proliferation and activation of T cells and to induce apoptosis of neurons and oligodendrocytes, and seems to be implicated in autoimmune diseases. Thus, *TRAIL* and *TRAIL receptor* genes are potential candidates for involvement in susceptibility to multiple sclerosis (MS). To test whether single-nucleotide polymorphisms (SNPs) in the human genes encoding *TRAIL*, *TRAILR-1*, *TRAILR-2*, *TRAILR-3* and *TRAILR-4* are associated with MS susceptibility, we performed a candidate gene case-control study in the Spanish population. 59 SNPs in the *TRAIL* and *TRAIL receptor* genes were analysed in 628 MS patients and 660 controls, and validated in an additional cohort of 295 MS patients and 233 controls. Despite none of the SNPs withstood the highly conservative Bonferroni correction, three SNPs showing uncorrected p values<0.05 were successfully replicated: rs4894559 in *TRAIL* gene, p = 9.8×10^−4^, OR = 1.34; rs4872077, in *TRAILR-1* gene, p = 0.005, OR = 1.72; and rs1001793 in *TRAILR-2* gene, p = 0.012, OR = 0.84. The combination of the alleles G/T/A in these SNPs appears to be associated with a reduced risk of developing MS (p = 2.12×10^−5^, OR = 0.59). These results suggest that genes of the TRAIL/TRAIL receptor system exerts a genetic influence on MS.

## Introduction

Multiple Sclerosis (MS) is a chronic, inflammatory, demyelinating and neurodegenerative disease of the Central Nervous System (CNS). Although its aetiology still remains partially unknown, MS is thought to arise from complex interactions of both environmental and genetic factors.

Linkage and association studies demonstrate that MS follows a polygenic trait, with susceptibility being determined by different genes, each exerting a relatively moderate effect on overall disease predisposition [1]. For decades, the only candidate genes consistently corroborated as influencing MS susceptibility were encoded within the Major Histocompatibility Complex region (MHC, HLA) [2]–[4]. Nevertheless, although data from the latest large-scale association studies strongly support the dominance of the HLA locus in genetic susceptibility to MS, they have also led to substantial progress in unravelling the involvement of other genes outside the HLA region [5]–[10].

The tumour necrosis factor (TNF) family genes are strong candidates for involvement in MS risk because they play important roles in the interaction between the CNS and the immune system in both defence and apoptosis of neurons and glial cells in neuroinflammatory diseases. The TNF-related apoptosis-inducing ligand (TNFSF10/TRAIL) [11] is a type II transmembrane protein belonging to the TNF/nerve growth factor superfamily [12] capable of inducing apoptosis in susceptible cells through interaction with its receptors TNFRSF10A/TRAILR-1 and TNFRSF10B/TRAILR-2. Two other cell-bound receptors - TNFRSF10C/TRAILR-3 and TNFRSF10D/TRAILR-4 - and a soluble receptor – Osteoprotegerin - do not contain functional death domains and act as decoy receptors for TRAIL.

The TRAIL/TRAIL receptor system might be playing an important role in the pathogenesis of MS, because of the expression of apoptosis-mediating and apoptosis-blocking TRAIL receptors [13], but its exact function is just beginning to unfold. On one hand, TRAIL might exert a neurotoxic role, as activated brain macrophages and microglia both express TRAIL [14], and might interact with TRAIL receptors expressed in neurons, oligodendrocytes and astrocytes, causing inflammation [15], cell injury or death [16]. In this sense, it has been reported that both oligodendrocytes [17], and neurons [14] are susceptible to TRAIL induced apoptosis.

On the other hand, TRAIL blockade with the administration of soluble rTRAIL receptor, during the effector phase of MOG-induced experimental autoimmune encephalomyelitis (EAE), enhances the formation of inflammatory lesions and the extent of demyelination in the CNS, and exacerbated EAE, suggesting that TRAIL may be inhibiting autoimmune inflammation in the CNS [18]. However, the precise mechanism whereby TRAIL exerts this mechanism *in vivo* is not clear. It may inhibit activation of autoreactive T cells that initiate autoimmune responses *in vitro* and *in vivo* [19] or may induce apoptosis of inflammatory cells. Recently, it has been reported that activation of T cells with IL-2 resulted in TRAIL-mediated death of antigen-specific memory CD8+ T cells and that human T helper (Th) 1 cell clones are sensitive to TRAIL-induced apoptosis, whereas Th2 cell lines are not. Whether this is also true *in vivo* remains to be clarified [20].

In conclusion, some studies detected anti-inflammatory properties of TRAIL showing neuroprotective potential, while others considered it as a mediator of cell damage and inflammatory response. As TRAIL might be acting in both ways, as a “double-edge sword” [21], [22], *TRAIL* and *TRAIL receptor* genes are potential candidates for involvement in the development of multiple sclerosis.

The aim of the present study was to examine the potential role of polymorphisms in the genes encoding *TRAIL* and its four surface receptors on MS susceptibility, and to search for disease-associated combinations of allelic variants in these polymorphisms.

## Results

Genotype frequencies and P values for the 59 polymorphisms under the four different genetic models were calculated. Three SNPs were discarded from the study because of technical problems in the manufacturing process (rs3136597, rs13257094 and rs4242387). Genotype distribution of the remaining 56 SNPs in controls and MS patients in the original and in the validation cohorts is shown in Table S1. There were no statistically significant differences in the genotyping success rate between cases and controls for any of these markers. Overall, the results were in Hardy- Weinberg equilibrium after adjustment by the Benjamini and Hochberg method (Table S2), with the exceptions of rs3181143 in the *TRAIL* gene and rs12545733 in the *TRAILR-3* gene, both in MS patients and in controls. Thus, these two polymorphisms were also discarded from further analyses.

After applying the highly conservative Bonferroni correction for multiple tests, none of the SNPs analyzed remained significantly associated with MS risk in the original cohort. However, a total of six SNPs (rs4894559, rs1001793, rs3924519, rs11779484, rs4460370 and rs9314261) showed nominally significant association in different genetics models, and rs4872077 showed a marked trend under a recessive model (p = 0.052) as shown in Table 1, hence, these seven SNPs could not be completely dismissed.

**Table 1:** Genotype frequencies of the TRAIL and TRAILR significant polymorphisms.

SNP ID	Model	Original Cohort		Validation Cohort		Joint Analysis	
		p value	OR (95% C.I.)	p value	OR (95% C.I.)	p value	OR (95% C.I.)
rs4894559	L-A [GG = 0, GA = 1, AA = 2]	**0.015**	**1.29 (1.05–1.58)**	**0.030**	**1.44 (1.03–2.00)**	**9.8×10^−4^**	**1.34 (1.12–1.59)**
	C [GA *vs.* GG]	**0.014**	**1.15 (0.89–1.47)**	**0.022**	**1.70 (1.16–2.48)**	**0.004**	**1.31 (1.06–1.61)**
	C [AA *vs.* GG]	**0.014**	**2.47 (1.29–4.72)**	**0.022**	**0.96 (0.33–2.79)**	**0.004**	**1.93 (1.12–3.34)**
	D [(GA+AA) *vs.* GG]	0.073	1.24 (0.98–1.58)	**0.010**	**1.62 (1.12–2.35)**	**0.002**	**1.36 (1.11–1.66)**
	R [AA *vs.* (GG+GA)]	**0.006**	**2.37 (1.25–4.52)**	p>0.1		**0.034**	**1.78 (1.03–3.06)**
rs4872077	L-A [TT = 0, TC = 1, CC = 2]	p>0.1		**0.047**	**1.32 (1.00–1.74)**	0.097	1.14(0.98–1.32)
	C [TC *vs.* TT]	p>0.1		p>0.1		**0.021**	**0.99 (0.81–1.20)**
	C [CC *vs.* TT]	p>0.1		p>0.1		**0.021**	**1.71 (1.15–2.55)**
	D [(TC+CC) *vs.* TT]	p>0.1		p>0.1		p>0.1	
	R [CC *vs.* (TT+TC)]	0.052	1.60 (0.99–2.58)	0.060	1.88 (0.95–3.71)	**0.005**	**1.72 (1.17–2.54)**
rs11779484	L-A [TT = 0, TC = 1, CC = 2]	0.069	0.75 (0.54–1.02)	p>0.1			
	C [TC *vs.* TT]	**0.046**	**0.69 (0.49–0.96)**	p>0.1			
	C [CC *vs.* TT]	**0.046**	**3.01 (0.31–29.03)**	p>0.1			
	D [(TC+CC) *vs.* TT]	**0.039**	**0.71 (0.51–0.99)**	p>0.1			
	R [CC *vs.* (TT+TC)]	p>0.1		p>0.1			
rs1001793	L-A [GG = 0, GA = 1, AA = 2]	**0.040**	**0.84 (0.71–0.99)**	p>0.1		**0.012**	**0.84 (0.73–0.96)**
	C [GA *vs.* GG]	p>0.1		p>0.1		**0.035**	**0.80 (0.66–0.98)**
	C [AA *vs.* GG]	p>0.1		p>0.1		**0.035**	**0.73 (0.53–0.99)**
	D [(GA+AA) *vs.* GG]	0.074	0.82 (0.65–1.02)	0.064	0.72 (0.51–1.02)	**0.012**	**0.79 (0.65–0.95)**
	R [AA *vs.* (GG+GA)]	p>0.1		p>0.1		p>0.1	
rs4460370	L-A [CC = 0, CT = 1, TT = 2]	**0.026**	**1.21 (1.02–1.44)**	p>0.1			
	C [CT *vs.* CC]	**0.048**	**1.11 (0.88–1.40)**	p>0.1			
	C [TT *vs.* CC]	**0.048**	**1.64 (1.10–2.43)**	p>0.1			
	D [(CT+TT) *vs.* CC]	p>0.1		p>0.1			
	R [TT *vs.* (CC+CT)]	**0.021**	**1.56 (1.07–2.27)**	p>0.1			
rs9314261	L-A [GG = 0, GA = 1, AA = 2]	**0.026**	**0.78 (0.62–0.97)**	p>0.1			
	C [GA *vs.* GG]	0.067	0.74 (0.56–0.97)	**0.047**	**1.30 (0.87–1.95)**		
	C [AA *vs.* GG]	0.067	0.72 (0.37–1.40)	**0.047**	**0.43 (0.18–1.04)**		
	D [(GA+AA) *vs.* GG]	**0.020**	**0.73 (0.57–0.95)**	p>0.1			
	R [AA *vs.* (GG+GA)]	p>0.1		**0.035**	**0.40 (0.17–0.96)**		
rs3924519	L-A [TT = 0, TC = 1, CC = 2]	p>0.1		p>0.1			
	C [TC *vs.* TT]	**0.021**	**1.06 (0.84–1.34)**	p>0.1			
	C [CC *vs.* TT]	**0.021**	**0.61 (0.41–0.90)**	p>0.1			
	D [(TC+CC) *vs.* TT]	p>0.1		p>0.1			
	R [CC *vs.* (TT+TC)]	**0.006**	**0.59 (0.40–0.87)**	p>0.1			

Abbreviations: L-A: Log-Additive model; C: Codominant model; D: Dominant model; R: Recessive model; SNP ID: SNP identification; OR (95% CI): odds ratio with confidence interval at 95%.

The log-additive model is the equivalent to calculate the odds ratio for the minor allele. The codominant model compared the homozygous genotype for the most frequent allele to the heterozygous and to the other homozygous genotype. The dominant model compared the homozygous genotype for the most frequent allele to the combination of the heterozygous and the other homozygous genotype. The recessive model compared a combination of the homozygous for the most frequent allele and the heterozygous genotypes to the homozygous for the minor allele.

Values in bold indicates p values lower than 0.05; Joint analysis data from non replicated associations are not shown.

The validation with a second cohort of patients and healthy controls ended with only four out of these seven SNPs showing p values lower than 0.05. Furthermore, rs9314261 showed the opposite allele associated in the replication cohort as compared to the original study, therefore only three associations were successfully replicated: rs4894559 in *TRAIL*, rs4872077 in *TRAILR-1* and rs1001793 in *TRAILR-2*.

When both cohorts were combined in order to increase the sample size and so, gain statistical power, statistical significance increased compared to that observed in each cohort separately, being the lowest p values for each SNPs as follows: rs4894559, risk allele = A, additive model, p = 9.8×10^−4^, OR = 1.34; rs4872077, risk allele = C, recessive model, p = 0.005, OR = 1.72; and rs1001793, protective allele = A, additive model, p = 0.012, OR = 0.84.

We examined the impact of allelic combinations of the three previously described SNPs on susceptibility to MS. Analysis of all possible combinations highlighted rs4894559G, rs4872077T and rs1001793A as the most relevant combination which was significantly underrepresented in MS patients and displayed protective effects for MS. This association was present in the original cohort (p = 2.2×10^−4^, OR = 0.59) as well as in the validation cohort (p = 0.0426, OR = 0.62). Both cohorts combined yielded p = 2.12×10^−5^, OR = 0.59. Distribution of carriers of this allelic combination in the original cohort, validation cohort and in the joint analysis is shown in Table 2.

**Table 2:** Distribution of the carriers of rs4894559G/rs4872077T/rs1001793A.

	Original Cohort		Validation Cohort		Joint Analysis	
	MS patients	Controls	MS patients	Controls	MS patients	Controls
GTA carriers	270 (44.3%)	349 (54.1%)	116 (41.7%)	121 (53.8%)	386 (43.5%)	470 (54.0)
Non GTA carriers	339 (55.7%)	296 (45.9%)	162 (58.3%)	104 (46.2%)	501 (56.5%)	400 (46.0)

GTA carriers are made up for subjects with genotypes GG or GA in rs4894559, TT or TC in rs4872077 and AA or AG in rs1001793. Non GTA carriers are made up for subjects with genotypes AA in rs4894559, CC in rs4872077 and GG in rs1001793.

## Discussion

This is to the best of our knowledge the first study assessing the potential role of polymorphisms in the *TRAIL receptor* genes on MS susceptibility and also the first one undertaking an in-depth analysis of the SNPs in the *TRAIL* gene in this disease.

The TRAIL/TRAIL receptor system has been reported to participate in crucial steps in immune cell activation, migration, proliferation and differentiation and it seems to be implicated in a variety of autoimmune diseases [18], [19], [23]–[25]. TRAIL usually does not induce apoptosis in activated T cells, but it is capable of directly inhibit proliferation, activation and IFN-γ/IL-4 production of human T cells via blockade of calcium influx [26] and induce apoptosis of neurons and oligodendrocytes [16]. Thus, *TRAIL* and *TRAIL receptor* genes are potential candidates for involvement in the development of multiple sclerosis.

A previous study found an association between rs1131579 in the 3′-untranslated region of exon 5 in *TRAIL* and MS in a Japanese population [27]. However, we were unable to replicate this association in the Spanish population, as it was not polymorphic (frequency of the homozygous common genotype was 99.9% in controls and 99.9% in MS patients in the joint Spanish analysis). A previous study identified a highly polymorphic region in the TRAIL promoter but could not detect any associations of the SNPs in this region with MS susceptibility [28]. In our study, we found seven candidate SNPs associated with MS risk in the original cohort and carried out a validation study in a second cohort. This allowed us to confirm the associations of three of them (rs4894559, risk allele = A; rs4872077, risk allele = C; and rs1001793, protective allele = A), and the joint analysis gave us a more reliable measure of the magnitude of these associations. Furthermore, the study of allele combinations within these three polymorphisms showed a stronger association, being the combination of G, T and A alleles for rs4894559, rs4872077 and rs1001793 respectively, a protective factor for MS. This suggests that these polymorphisms may be acting together and may have a synergic effect on MS susceptibility.

It is unclear whether the lack of validation for the remaining 4 SNPs was due to a lack of statistical power or else they were false positives, although genotype frequencies seem to support that they were definitively false positives, with the exception of rs11779484 in *TRAILR-1* (Table S1).

The SNP rs4894559 is located in the *TRAIL* gene, 580 nucleotides upstream of exon 3, SNP rs4872077 is located in the *TRAILR-1* gene, 12 nucleotides downstream of exon 5 and rs1001793 is located in the *TRAILR-2* gene, 92 nucleotides upstream of exon 2. This latter SNP is a proxy of rs1001792 (r^2^ = 0.91), located 345 nucleotides upstream of exon 2.

All TRAIL apoptotic and decoy receptors are expressed in the human brain in microvascular endothelial cells [29], oligodendrocytes, neurons and astrocytes [30], while TRAIL is mainly expressed in brain macrophages and infiltrating leukocytes. As the TRAIL system is characterised by a complex panel of receptors leading to highly variable effects of TRAIL signalling dependent on microenvironment, cell type and timing [21], the interaction of the genes included in this allele combination may lead to a deregulation of inflammatory pathways. We hypothesize that the susceptibility conferred by these SNPs could be due to a change of a protein binding site in the DNA sequence, which may alter mechanisms involved in alternative splicing, or a change in the expression levels of *TRAIL* and its death receptors. These alterations may affect the TRAIL system, resulting in a more favourable anti-inflammatory microenvironment. In support of these mechanisms, some authors have reported that balance between death and decoy receptors may determine whether a cell could or could not undergo *TRAIL*-mediated apoptosis [17], [30]. Thus, this triallelic combination might result in target cells, such as oligodendrocytes, remaining resistant to TRAIL-induced apoptosis, which might be one of the putative cytotoxic effector mechanisms in MS pathogenesis.

In conclusion, the association of these genes with MS risk supports a biological role for the *TRAIL* and *TRAIL receptor* gene products in the pathogenesis of MS. Therefore, the functional consequences of these SNPs need to be clarified, as separately they might be playing only a minor effect on MS susceptibility but the sum or interaction of multiple risk alleles may represent a key contributor to the overall MS susceptibility.

## Materials and Methods

### Ethics Statement

Written informed consent was obtained from patients and controls. The study was approved by the Institutional Research Ethics Committees of the respective hospitals (Comisión de Ética y de Investigación del Hospital Regional Universitario Carlos Haya, Comité Ético del Hospital Universitario 12 de Octubre de Madrid, Comité Ético de Investigación Clínica del Hospital Clínico San Carlos and Comité Ético de Investigación Clínica del Instituto de Investigación Sanitaria San Carlos).

### Study subjects

A total of 628 patients were recruited for the original cohort through the Multiple Sclerosis unit of Carlos Haya Regional University Hospital in Malaga, Spain. As controls, 660 sex and age-matched healthy unrelated subjects were obtained from the Malaga Blood Bank. For the validation cohort, we selected a total of 295 patients from the 12 de Octubre University Hospital (n = 59) and San Carlos Clinic Hospital (n = 236), both in Madrid, Spain, and 233 healthy subjects from San Carlos Clinic Hospital. All patients in both cohorts were Spanish Caucasian individuals and fulfilled the McDonald criteria [31] for MS diagnosis.

The following demographic and clinical characteristics of the MS patients were assessed: sex, age, age at onset, clinical form at onset and at present, disease duration, expanded disability status scale (EDSS) score, and progression index (current EDSS score/disease duration). These clinical characteristics of the MS patients are summarised in Table 3.

**Table 3:** Demographic and clinical characteristics of the 923 MS patients.

Characteristics	Original cohort	Validation cohort
Gender (%):		
Female	431 (68.6%)	192 (65.1%)
Male	197 (31.4%)	103 (34.9%)
Age (years)	43.50±11.35 (15–77)	40.64±9.00 (19–72)
Mean age at onset (years)	29.59±9.73 (4–68)	28.90±7.76 (8–53)
Clinical form at onset:		
Relapsing	535 (99.4%)	259 (99.6%)
Progressive	3 (0.6%)	1 (0.4%)
Clinical form at present		
RR	422 (78.4%)	244 (93.8%)
SP	113 (21.0%)	15 (5.8%)
PP	3 (0.6%)	1 (0.4%)
Mean disease duration (years)	14.5±8.1 (1–41)	12.18±7.28 (0–49)
Current EDSS score	2.84±2.26 (0–9)	2.43±1.68 (0–7)
Progression index (current EDSS score/disease duration)	0.23±0.24	0.18±0.12

Quantitative data are presented as mean ± standard deviation (minimum–maximum).

EDSS Expanded disability status scale, PP primary progressive, PR progressive relapsing, RR relapsing–remitting, SP secondary progressive.

### SNP identification and selection

54 Tag-SNPs spanning the following genes: *TRAIL* (mapped at chromosome 3q26) and its four receptors *TRAILR-1*, *TRAILR-2*, *TRAILR-3* and *TRAILR-4* (mapped at chromosome 8p21-22), were selected using the web tool “SYSNPs” (www.sysnps.org). Flanking regions of 2000 nucleotides upstream and 500 nucleotides downstream were included. A minor allele frequency of at least 0.1 and a minimum r^2^ coefficient of 0.8 were used to select Tag-SNPs. In addition, we also selected five exonic SNPs in the *TRAIL* gene, corresponding to rs6763816 and rs11545817 in exon 1, rs16845759 in exon 2, rs4491934 in exon 3 and rs1131579 in the 3′ UTR of exon 5 (the latter has previously been reported to be a high risk factor for MS in a Japanese population) [27].

### Genotyping

Genomic DNA was extracted from peripheral blood nucleated cells using the Genomic DNA Purification Kit® (Gentra Systems Inc, Minneapolis, MN, USA).

All polymorphisms were genotyped using TaqMan assays (AppliedBiosystems, Inc., Foster City, CA, USA) and the OpenArray Platform (BioTrove, Woburn, MA, USA) following the protocols recommended by manufacturers. In short, reactions were performed in 3072 through-hole arrays under the following conditions: 93°C for 10 minutes, followed by 50 cycles of 95°C for 45 seconds, 94°C for 13 seconds and 53°C for 134 seconds. The median DNA concentration used was 50 ng/µL.

### Statistical analysis

Statistical analysis was performed using the SPSS software (version 11.5.1) and the SNPassoc R package (R software version 2.10.0) [32].

Deviations from Hardy-Weinberg Equilibrium were tested using an exact test as Wigginton *et al.* [33]. Multiple testing corrections were carried out using the Benjamini and Hochberg False Discovery Rate [34].

To test if any individual SNP was associated with MS susceptibility, genotype frequencies were compared using a likelihood ratio test under four different genetic models (Codominant, Dominant, Recessive and Additive). P values lower than 0.05 were considered to be statistically significant. Logistic regression models were used to estimate crude odds ratios (ORs) and 95% confidence intervals (95% CI). To avoid false-positive results due to multiple testing we applied the Bonferroni correction that is robust against positive dependence.

The statistical power was calculated with QUANTO 1.2.4 (http://hydra.usc.edu/gxe). With the original cohort, we had a 91.52% power to detect, at a significance level of 0.05, an OR effect size of 1.5 when the minor allele frequency was 0.1 under a log-additive model, decreasing to 50.41% to detect the same effect at a Bonferroni level (p = 0.00089). Power in the validation cohort, taking into account the results from the original cohort ranged from 26 to 60% (Table S3).

To study allelic combinations and test their effects on MS susceptibility we chose those SNPs with a replicated significantly different distribution between MS patients and controls. Allelic combinations frequencies were estimated using the Expectation-Maximization algorithm, and P values and ORs with 95% CI were calculated with a General Linear Model regression.

## Supporting Information

Table S1 Abbreviations: SNP ID, SNP identification; Chr, chromosome; 1>2, major>minor allele; NSC: Non Synonymous Coding.
(DOC)

Table S2 Abbreviations: SNP ID, SNP identification; P_BH_, P values correct with Benjamini and Hochberg. Departures from Hardy-Weinberg Equilibrium were tested using an exact test as Wigginton et al. P values were corrected for multiple testing using the Benjamini and Hochberg method. Values in bold indicates a deviation from the Hardy-Weinberg equilibrium. A hyphen indicates that the Hardy-Weinberg Equilibrium could not be calculated due to the lack of one or two genotypes.
(DOC)

Table S3 Abbreviations: SNP ID, SNP identification; OR = odds ratio to be replicated.
(DOC)
