# Supplementary material for: TRAIL/TRAIL Receptor System and Susceptibility to Multiple Sclerosis
Source: PLoS One. 2011 Jul 21;6(7):e21766. doi: 10.1371/journal.pone.0021766 (PMC3140982; doi:10.1371/journal.pone.0021766)
Supplement: Table S1 — Abbreviations: SNP ID, SNP identification; Chr, chromosome; 1>2, major>minor allele; NSC: Non Synonymous Coding. (DOC) [file pone.0021766.s001.doc]

**Table S1. Genotypes Frequencies obtained from Original and Validation cohorts.**

| SNP ID | Gene | Chr. | Location | Allele  1 > 2 | Original Cohort (Málaga) | | | | | | Validation Cohort (Madrid) | | | | | |
| --- | --- | --- | --- | --- | --- | --- | --- | --- | --- | --- | --- | --- | --- | --- | --- | --- |
|  |  |  |  |  | Controls (n = 660) (%) | | | MS patients (n = 628) (%) | | | Controls (n = 233) (%) | | | MS patients (n = 295) (%) | | |
|  |  |  |  |  | 11 | 12 | 22 | 11 | 12 | 22 | 11 | 12 | 22 | 11 | 12 | 22 |
| rs3181143 | TRAIL | 3 | Intronic | C>A | 74 (11.8%) | 484 (77.3%) | 68 (10.9%) | 97 (17.0%) | 424 (74.1%) | 51 (8.9%) | 51 (24.5%) | 146 (70.2%) | 11 (5.3%) | 41 (15.2%) | 205 (75.9%) | 24 (8.9%) |
| rs3136594 | TRAIL | 3 | Intronic | C>T | 277 (43.7%) | 281 (44.3%) | 76 (12.0%) | 260 (42.2%) | 297 (48.2%) | 59 (9.6%) | 92 (42.6%) | 101 (46.8%) | 23 (10.6%) | 120 (42.3%) | 133 (46.8%) | 31 (10.9%) |
| rs4894559 | TRAIL | 3 | Intronic | G>A | 461 (71.0%) | 174 (26.8%) | 14 (2.2%) | 400 (66.3%) | 173 (28.7%) | 30 (3.5%) | 160 (70.2%) | 61 (26.8%) | 7 (3.1%) | 167 (59.2%) | 108 (38.3%) | 7 (2.5%) |
| rs231983 | TRAIL | 3 | Intronic | A>C | 330 (50.2%) | 274 (41.6%) | 54 (8.2%) | 305 (48.6%) | 269 (42.9%) | 53 (8.5%) | 124 (53.9%) | 91 (39.6%) | 15 (6.5%) | 132 (45.2%) | 130 (44.5%) | 30 (10.3%) |
| rs179777 | TRAIL | 3 | Intronic | A>G | 454 (69.7%) | 183 (28.1%) | 14 (2.2%) | 446 (71.9%) | 167 (26.9%) | 7 (1.1%) | 165 (72.4%) | 60 (26.3%) | 3 (1.3%) | 205 (72.2%) | 70 (24.6%) | 9 (3.2%) |
| rs3136581 | TRAIL | 3 | 5' Upstream | C>T | 418 (63.6%) | 211 (32.1%) | 28 (4.3%) | 391 (62.7%) | 205 (32.9%) | 28 (4.5%) | 131 (57.0%) | 84 (36.5%) | 15 (6.5%) | 197 (67.7%) | 80 (27.5%) | 14 (4.8%) |
| rs6763816 | TRAIL | 3 | Exon 1, NSC | C>T | 656 (99.5%) | 3 (0.5%) | 0 (0.0%) | 616 (99.5%) | 3 (0.5%) | 0 (0.0%) | 226 (100%) | 0 (0.0%) | 0 (0.0%) | 287 (100%) | 0 (0.0%) | 0 (0.0%) |
| rs16845759 | TRAIL | 3 | Exon 2, NSC | G>T | 648 (98.8%) | 8 (1.2%) | 0 (0.0%) | 611 (97.9%) | 13 (2.1%) | 0 (0.0%) | 227 (99.6%) | 1 (0.4%) | 0 (0.0%) | 277 (97.5%) | 7 (2.5%) | 0 (0.0%) |
| rs4491934 | TRAIL | 3 | Exon 3, NSC | G>A | 644 (99.8%) | 1 (0.2%) | 0 (0.0%) | 604 (99.7%) | 2 (0.3%) | 0 (0.0%) | 230 (99.1%) | 2 (0.9%) | 0 (0.0%) | 277 (98.9%) | 3 (1.1%) | 0 (0.0%) |
| rs1823227 | TRAIL | 3 | Intronic | T>G | 288 (43.6%) | 297 (45.0%) | 75 (11.4%) | 272 (43.9%) | 281 (45.3%) | 67 (10.8%) | 95 (41.3%) | 111 (48.3%) | 24 (10.4%) | 131 (45.0%) | 132 (45.4%) | 28 (9.6%) |
| rs3136587 | TRAIL | 3 | Intronic | T>C | 473 (72.4%) | 165 (25.3%) | 15 (2.3%) | 471 (75.2%) | 140 (22.4%) | 15 (2.4%) | 180 (78.3%) | 48 (20.9%) | 2 (0.9%) | 215 (73.6%) | 70 (24.0%) | 7 (2.4%) |
| rs1131579 | TRAIL | 3 | Exon 5, 3´UTR | G>A | 638 (100%) | 0 (0.0%) | 0 (0.0%) | 586 (99.8%) | 1 (0.2%) | 0 (0.0%) | 225 (99.6%) | 1 (0.4%) | 0 (0.0%) | 274 (100%) | 0 (0.0%) | 0 (0.0%) |
| rs11545817 | TRAIL | 3 | Exon 1, NSC | G>A | 658 (100%) | 0 (0.0%) | 0 (0.0%) | 626 (100%) | 0 (0.0%) | 0 (0.0%) | 226 (100%) | 0 (0.0%) | 0 (0.0%) | 274 (100%) | 0 (0.0%) | 0 (0.0%) |
| rs2230229 | TRAILR-1 | 8 | Exon 10, NSC | A>G | 475 (73.3%) | 165 (25.5%) | 8 (1.2%) | 441 (71.9%) | 164 (26.8%) | 8 (1.3%) | 162 (71.1%) | 59 (25.9%) | 7 (3.1%) | 213 (72.9%) | 73 (25.0%) | 6 (2.1%) |
| rs11775256 | TRAILR-1 | 8 | Intronic | C>T | 372 (57.1%) | 237 (36.3%) | 43 (6.6%) | 365 (59.9%) | 212 (34.8%) | 32 (5.3%) | 143 (65.3%) | 69 (31.5%) | 7 (3.2%) | 175 (61.0%) | 94 (32.8%) | 18 (6.3%) |
| rs11780345 | TRAILR-1 | 8 | Intronic | T>C | 287 (45.2%) | 283 (44.6%) | 65 (10.2%) | 280 (45.4%) | 268 (43.4%) | 69 (11.2%) | 115 (50.2%) | 98 (42.8%) | 16 (7.0%) | 140 (48.1%) | 121 (41.6%) | 30 (10.3%) |
| rs6557627 | TRAILR-1 | 8 | Intronic | C>G | 450 (68.9%) | 180 (27.6%) | 23 (3.5%) | 418 (67.3%) | 181 (29.1%) | 22 (3.5%) | 174 (75.3%) | 50 (21.6%) | 7 (3.0%) | 190 (66.4%) | 83 (29.0%) | 13 (4.5%) |
| rs2235126 | TRAILR-1 | 8 | Intronic | C>T | 324 (49.7%) | 279 (42.8%) | 49 (7.5%) | 297 (48.0%) | 275 (44.4%) | 47 (7.6%) | 103 (44.6%) | 99 (42.9%) | 29 (12.6%) | 146 (50.3%) | 120 (41.4%) | 24 (8.3%) |
| rs10097540 | TRAILR-1 | 8 | Intronic | C>A | 371 (58.0%) | 246 (38.4%) | 23 (3.6%) | 370 (61.8%) | 203 (33.9%) | 26 (4.3%) | 133 (58.3%) | 86 (37.7%) | 9 (3.9%) | 180 (64.5%) | 86 (30.8%) | 13 (4.7%) |
| rs4872077 | TRAILR-1 | 8 | Intronic | T>C | 359 (54.9%) | 265 (40.5%) | 30 (4.6%) | 342 (55.4%) | 231 (37.1%) | 44 (7.1%) | 132 (57.6%) | 84 (36.7%) | 13 (5.7%) | 145 (50.9%) | 111 (38.9%) | 29 (10.2%) |
| rs20576 | TRAILR-1 | 8 | Exon 5, NSC | A>C | 387 (58.9%) | 233 (35.5%) | 37 (5.6%) | 371 (59.4%) | 221 (35.4%) | 33 (5.3%) | 148 (64.9%) | 67 (29.4%) | 13 (5.7%) | 166 (57.8%) | 105 (36.6%) | 16 (5.6%) |
| rs4242392 | TRAILR-1 | 8 | Intronic | T>C | 402 (61.2%) | 213 (32.4%) | 42 (6.4%) | 372 (59.7%) | 215 (34.5%) | 36 (5.8%) | 121 (52.4%) | 100 (43.3%) | 10 (4.3%) | 179 (61.3%) | 99 (33.9%) | 14 (4.8%) |
| rs6995408 | TRAILR-1 | 8 | Intronic | G>A | 163 (25.3%) | 330 (51.2%) | 152 (23.6%) | 142 (23.7%) | 310 (51.8%) | 147 (24.5%) | 41 (19.2%) | 111 (51.9%) | 62 (29.0%) | 70 (24.9%) | 153 (54.4%) | 58 (20.6%) |
| rs4526369 | TRAILR-1 | 8 | Intronic | A>G | 365 (56.2%) | 249 (38.4%) | 35 (5.4%) | 336 (54.3%) | 248 (40.1%) | 35 (5.7%) | 123 (53.9%) | 85 (37.3%) | 20 (8.8%) | 170 (58.2%) | 104 (35.6%) | 18 (6.2%) |
| rs11785328 | TRAILR-1 | 8 | Intronic | C>T | 360 (54.7%) | 259 (39.4%) | 39 (5.9%) | 330 (53.0%) | 250 (40.1%) | 43 (6.9%) | 129 (55.8%) | 90 (39.0%) | 12 (5.2%) | 151 (53.2%) | 113 (39.8%) | 20 (7.0%) |
| rs13255394 | TRAILR-1 | 8 | 5' Upstream | T>C | 172 (31.5%) | 247 (45.2%) | 127 (23.3%) | 163 (30.6%) | 231 (43.3%) | 139 (26.1%) | 39 (17.4%) | 128 (57.1%) | 57 (25.4%) | 54 (19.1%) | 160 (56.5%) | 69 (24.4%) |
| rs11779484 | TRAILR-1 | 8 | Intronic | T>C | 553 (84.8%) | 98 (15.0%) | 1 (0.2%) | 551 (88.7%) | 67 (10.8%) | 3 (0.5%) | 196 (85.2%) | 31 (13.5%) | 3 (1.3%) | 255 (87.3%) | 34 (11.6%) | 3 (1.0%) |
| rs6557628 | TRAILR-1 | 8 | Intronic | T>G | 425 (65.7%) | 199 (30.8%) | 23 (3.6%) | 405 (65.7%) | 183 (29.7%) | 28 (4.5%) | 144 (64.0%) | 73 (32.4%) | 8 (3.6%) | 198 (70.5%) | 76 (27.0%) | 7 (2.5%) |
| rs1047275 | TRAILR-2 | 8 | 3' UTR | C>G | 187 (28.5%) | 351 (53.4%) | 119 (18.1%) | 170 (27.3%) | 327 (52.5%) | 126 (20.2%) | 69 (30.3%) | 111 (48.7%) | 48 (21.1%) | 79 (27.1%) | 141 (48.5%) | 71 (24.4%) |
| rs6557609 | TRAILR-2 | 8 | Intronic | C>T | 493 (75.0%) | 150 (22.8%) | 14 (2.1%) | 478 (76.6%) | 134 (21.5%) | 12 (1.9%) | 176 (76.9%) | 46 (20.1%) | 7 (3.1%) | 216 (75.3%) | 66 (23.0%) | 5 (1.7%) |
| rs7834266 | TRAILR-2 | 8 | Intronic | C>T | 242 (36.8%) | 328 (49.8%) | 88 (13.4%) | 250 (40.1%) | 284 (45.5%) | 90 (14.4%) | 94 (40.7%) | 107 (46.3%) | 30 (13.0%) | 115 (39.5%) | 137 (47.1%) | 39 (13.4%) |
| rs1001793 | TRAILR-2 | 8 | Intronic | G>A | 273 (41.9%) | 302 (46.3%) | 77 (11.8%) | 290 (46.8%) | 272 (43.9%) | 57 (9.2%) | 93 (40.4%) | 108 (47.0%) | 29 (12.6%) | 138 (48.6%) | 113 (39.8%) | 33 (11.6%) |
| rs13270480 | TRAILR-2 | 8 | Intronic | T>A | 327 (50.5%) | 269 (41.6%) | 51 (7.9%) | 313 (51.7%) | 248 (40.9%) | 45 (7.4%) | 108 (48.6%) | 94 (42.3%) | 20 (9.0%) | 155 (56.2%) | 105 (38.0%) | 16 (5.8%) |
| rs7843721 | TRAILR-2 | 8 | Intronic | G>T | 509 (77.7%) | 135 (20.6%) | 11 (1.7%) | 457 (73.9%) | 143 (23.1%) | 18 (2.9%) | 165 (72.1%) | 59 (25.8%) | 5 (2.2%) | 203 (73.0%) | 63 (22.7%) | 12 (4.3%) |
| rs4424253 | TRAILR-2 | 8 | Intronic | C>T | 455 (70.2%) | 178 (27.5%) | 15 (2.3%) | 447 (72.7%) | 150 (24.4%) | 18 (2.9%) | 161 (71.6%) | 59 (26.2%) | 5 (2.2%) | 197 (68.4%) | 79 (27.4%) | 12 (4.2%) |
| rs11135693 | TRAILR-2 | 8 | Intronic | C>A | 278 (42.5%) | 296 (45.3%) | 80 (12.2%) | 245 (39.5%) | 290 (46.7%) | 86 (13.8%) | 103 (45.2%) | 103 (45.2%) | 22 (9.6%) | 118 (40.5%) | 150 (51.5%) | 23 (7.9%) |
| rs4460370 | TRAILR-2 | 8 | Intronic | C>T | 316 (49.5%) | 271 (42.5%) | 51 (8.0%) | 269 (45.1%) | 256 (43.0%) | 71 (11.9%) | 93 (43.7%) | 95 (44.6%) | 25 (11.7%) | 130 (46.4%) | 117 (41.8%) | 33 (11.8%) |
| rs11135696 | TRAILR-3 | 8 | 5' UTR | G>A | 400 (62.6%) | 212 (33.2%) | 27 (4.2%) | 394 (63.7%) | 207 (33.4%) | 18 (2.9%) | 138 (61.9%) | 75 (33.6%) | 10 (4.5%) | 189 (65.4%) | 94 (32.5%) | 6 (2.1%) |
| rs4518666 | TRAILR-3 | 8 | Intronic | T>C | 304 (46.4%) | 287 (43.8%) | 64 (9.8%) | 291 (46.6%) | 272 (43.6%) | 61 (9.8%) | 101 (43.9%) | 103 (44.8%) | 26 (11.3%) | 124 (43.2%) | 123 (42.9%) | 40 (13.9%) |
| rs4872052 | TRAILR-3 | 8 | Intronic | T>C | 516 (78.5%) | 130 (19.8%) | 11 (1.7%) | 506 (81.2%) | 109 (17.5%) | 8 (1.3%) | 173 (74.9%) | 54 (23.4%) | 4 (1.7%) | 216 (74.0%) | 63 (21.6%) | 13 (4.5%) |
| rs4871846 | TRAILR-3 | 8 | Intronic | C>G | 272 (41.4%) | 301 (45.8%) | 84 (12.8%) | 267 (42.9%) | 276 (44.3%) | 80 (12.8%) | 84 (36.8%) | 106 (46.5%) | 38 (16.7%) | 105 (36.2%) | 131 (45.2%) | 54 (18.6%) |
| rs7008760 | TRAILR-3 | 8 | Intronic | C>G | 157 (24.4%) | 325 (50.5%) | 162 (25.2%) | 171 (28.5%) | 279 (46.4%) | 151 (25.1%) | 46 (20.5%) | 120 (53.6%) | 58 (25.9%) | 64 (23.4%) | 130 (47.6%) | 79 (28.9%) |
| rs12681513 | TRAILR-3 | 8 | Intronic | G>A | 487 (75.6%) | 149 (23.1%) | 8 (1.2%) | 434 (71.5%) | 167 (27.5%) | 6 (1.0%) | 149 (65.4%) | 73 (32.0%) | 6 (2.6%) | 204 (72.1%) | 69 (24.4%) | 10 (3.5%) |
| rs4077341 | TRAILR-3 | 8 | Intronic | T>G | 278 (42.4%) | 301 (46.0%) | 76 (11.6%) | 272 (43.5%) | 270 (43.1%) | 84 (13.4%) | 86 (37.6%) | 109 (47.6%) | 34 (14.8%) | 117 (40.2%) | 128 (44.0%) | 46 (15.8%) |
| rs12546238 | TRAILR-3 | 8 | Intronic | C>T | 535 (81.2%) | 109 (16.5%) | 15 (2.3%) | 506 (81.1%) | 111 (17.8%) | 7 (1.1%) | 189 (82.2%) | 39 (17.0%) | 2 (0.9%) | 228 (79.4%) | 56 (19.5%) | 3 (1.0%) |
| rs12545733 | TRAILR-3 | 8 | Intronic | C>T | 259 (42.7) | 341 (56.3%) | 6 (1.0%) | 286 (47.4%) | 313 (51.8%) | 5 (0.8%) | 98 (43.6%) | 119 (52.9%) | 8 (3.6%) | 136 (47.4%) | 149 (51.9%) | 2 (0.7%) |
| rs6557616 | TRAILR-3 | 8 | Exon 1, NSC | C>G | 378 (58.5%) | 241 (37.3%) | 27 (4.2%) | 377 (60.7%) | 223 (35.9%) | 21 (3.4%) | 142 (62.0%) | 78 (34.1%) | 9 (3.9%) | 174 (59.6%) | 111 (38.0%) | 7 (2.4%) |
| rs9314261 | TRAILR-3 | 8 | Intronic | G>A | 367 (66.7%) | 162 (29.5%) | 21 (3.8%) | 388 (73.2%) | 126 (23.8%) | 16 (3.0%) | 154 (69.7%) | 52 (23.5%) | 15 (6.8%) | 191 (67.5%) | 84 (29.7%) | 8 (2.8%) |
| rs7957 | TRAILR-4 | 8 | 3' UTR | T>C | 445 (67.9%) | 190 (29.0%) | 20 (3.1%) | 427 (68.4%) | 177 (28.4%) | 20 (3.2%) | 157 (68.0%) | 69 (29.9%) | 5 (2.2%) | 207 (71.1%) | 69 (23.7%) | 15 (5.2%) |
| rs7011559 | TRAILR-4 | 8 | Intronic | A>G | 443 (67.4%) | 194 (29.5%) | 20 (3.0%) | 428 (68.9%) | 174 (28.0%) | 19 (3.1%) | 160 (69.6%) | 63 (27.4%) | 7 (3.0%) | 192 (66.4%) | 86 (29.8%) | 11 (3.8%) |
| rs6557618 | TRAILR-4 | 8 | Intronic | T>A | 344 (52.8%) | 257 (39.5%) | 50 (7.7%) | 305 (49.4%) | 267 (43.2%) | 46 (7.4%) | 118 (52.0%) | 96 (42.3%) | 13 (5.7%) | 147 (51.8%) | 106 (37.3%) | 31 (10.9%) |
| rs1133782 | TRAILR-4 | 8 | Exon 7, NSC | C>T | 266 (40.6%) | 306 (46.7%) | 83 (12.7%) | 240 (38.8%) | 296 (47.8%) | 83 (13.4%) | 92 (40.2%) | 113 (49.3%) | 24 (10.5%) | 126 (43.0%) | 122 (41.6%) | 45 (15.4%) |
| rs3924519 | TRAILR-4 | 8 | Intronic | T>C | 319 (49.1%) | 252 (38.8%) | 79 (12.2%) | 305 (50.2%) | 256 (42.2%) | 46 (7.6%) | 100 (45.5%) | 95 (43.2%) | 25 (11.4%) | 133 (47.2%) | 118 (41.8%) | 31 (11.0%) |
| rs4871850 | TRAILR-4 | 8 | Intronic | A>G | 320 (48.9%) | 273 (41.7%) | 61 (9.3%) | 313 (50.2%) | 249 (40.0%) | 61 (9.8%) | 106 (46.1%) | 106 (46.1%) | 18 (7.8%) | 154 (53.7%) | 110 (38.3%) | 23 (8.0%) |
| rs7014131 | TRAILR-4 | 8 | Intronic | T>A | 411 (62.5%) | 217 (33.0%) | 30 (4.6%) | 384 (61.7%) | 204 (32.8%) | 34 (5.5%) | 122 (53.3%) | 98 (42.8%) | 9 (3.9%) | 189 (65.2%) | 87 (30.0%) | 14 (4.8%) |
| rs7462795 | TRAILR-4 | 8 | Intronic | C>T | 504 (76.9%) | 141 (21.5%) | 10 (1.5%) | 479 (77.3%) | 133 (21.5%) | 8 (1.3%) | 181 (78.7%) | 45 (19.6%) | 4 (1.7%) | 225 (79.8%) | 52 (18.4%) | 5 (1.8%) |

Abbreviations: SNP ID, SNP identification; Chr, chromosome; 1>2, major>minor allele; NSC: Non Synonymous Coding.
