# Supplementary material for: TRAIL/TRAIL Receptor System and Susceptibility to Multiple Sclerosis
Source: PLoS One. 2011 Jul 21;6(7):e21766. doi: 10.1371/journal.pone.0021766 (PMC3140982; doi:10.1371/journal.pone.0021766)
Supplement: Table S2 — Abbreviations: SNP ID, SNP identification; PBH, P values correct with Benjamini and Hochberg. Departures from Hardy-Weinberg Equilibrium were tested using an exact test as Wigginton et al. P values were corrected for multiple testing using the Benjamini and Hochberg method. Values in bold indicates a deviation from the Hardy-Weinberg equilibrium. A hyphen indicates that the Hardy-Weinberg Equilibrium could not be calculated due to the lack of one or two genotypes. (DOC) [file pone.0021766.s002.doc]

**Table S2. Departures from Hardy-Weinberg Equilibrium.**

| SNP ID | Original Cohort | | Validation Cohort | |
| --- | --- | --- | --- | --- |
|  | P_BH_ (Controls) | P_BH_ (MS) | P_BH_ (Controls) | P_BH_ (MS) |
| rs3181143 | **0** | **0** | **0** | **0** |
| rs3136594 | 1,4482 | 0,4648 | 1,2986 | 0,98609412 |
| rs4894559 | 1,42744 | 0,56784 | 1,36446154 | 0,2408 |
| rs231983 | 1,16097561 | 0,98591111 | 1,24262564 | 1,19213333 |
| rs179777 | 1,4616 | 0,48626667 | 1,34773333 | 0,74894815 |
| rs3136581 | 1,2260973 | 1,0542 | 1,31112258 | 0,57026667 |
| rs6763816 | - | - | - | - |
| rs16845759 | - | - | - | - |
| rs4491934 | - | - | - | - |
| rs1823227 | 1,15857778 | 0,98122927 | 1,25736471 | 1,00579394 |
| rs3136587 | 1,14292093 | 0,72644444 | 1,30795 | 1,01504 |
| rs1131579 | - | - | - | - |
| rs11545817 | - | - | - | - |
| rs2230229 | 0,80528 | 0,54523636 | 1,3888 | 1,14285714 |
| rs11775256 | 1,34603636 | 1,0376 | 1,28784444 | 0,79036364 |
| rs11780345 | 1,29475294 | 1,00142 | 1,376 | 1,02725 |
| rs6557627 | 1,3568 | 1,00873684 | 1,02666667 | 0,78516667 |
| rs2235126 | 1,27157333 | 0,69673333 | 1,41781818 | 1,19148936 |
| rs10097540 | 0,27216 | 1,07579574 | 1,2215 | 1,07221333 |
| rs4872077 | 0,33413333 | 0,98708235 | 1,36585366 | 0,80297778 |
| rs20576 | 1,18820513 | 1,12 | 1,0794 | 1,2173913 |
| rs4242392 | 0,45795556 | 0,93072 | 0,57773333 | 1,27272727 |
| rs6995408 | 1,25289231 | 0,89276923 | 1,41387826 | 0,6068 |
| rs4526369 | 1,3965 | 0,69244 | 1,204 | 1,0737641 |
| rs11785328 | 1,39557895 | 0,98344615 | 1,38856667 | 1,16666667 |
| rs13255394 | 0,3096 | **0,0448** | 0,4396 | 0,2536 |
| rs11779484 | 0,76486667 | 0,95822222 | 0,93072 | 0,60221538 |
| rs6557628 | 1,09803922 | 0,72210526 | 1,26544865 | 1,12 |
| rs1047275 | 0,3395 | 0,6909 | 1,30134118 | 0,99384444 |
| rs6557609 | 1,3472 | 0,9358 | 0,644 | 1,24444444 |
| rs7834266 | 0,80424615 | 0,959875 | 1,33333333 | 1,17261395 |
| rs1001793 | 1,38444444 | 0,97677576 | 1,24096 | 0,70889412 |
| rs13270480 | 1,35275 | 1,01573333 | 1,30232558 | 1,19102439 |
| rs7843721 | 1,291584 | 0,58408 | 1,27272727 | 0,21093333 |
| rs4424253 | 1,39132903 | 0,69110588 | 1,24444444 | 0,76184348 |
| rs11135693 | 1,134 | 1,09803922 | 1,25961379 | 0,13776 |
| rs4460370 | 1,3776 | 0,913024 | 1,09803922 | 0,831 |
| rs11135696 | 1,19148936 | 0,69353846 | 1,2173913 | 0,64288 |
| rs4518666 | 1,25936 | 1,03904348 | 1,19148936 | 0,8056 |
| rs4872052 | 1,45797647 | 0,88853333 | 1,16666667 | 0,19226667 |
| rs4871846 | 1,16666667 | 0,97096774 | 1,26709333 | 0,77028 |
| rs7008760 | 1,16626667 | 0,6055 | 1,23544615 | 0,90256552 |
| rs12681513 | 1,34288 | 0,315 | 1,51671579 | 0,6251 |
| rs4077341 | 1,40984828 | 0,70485333 | 1,14285714 | 0,77869474 |
| rs12546238 | 0,05973333 | 0,99816216 | 1,12 | 1,09803922 |
| rs12545733 | **0** | **0** | **0,0028** | **0** |
| rs6557616 | 0,75294545 | 0,58613333 | 1,24069474 | 0,21964444 |
| rs9314261 | 1,33466667 | 0,6728 | 0,05226667 | 1,17614 |
| rs7957 | 1,14285714 | 0,99285455 | 1,32885926 | 0,154 |
| rs7011559 | 1,13845455 | 1,01216744 | 1,3 | 1,03143158 |
| rs6557618 | 1,1816 | 0,7536 | 1,2468 | 0,48776 |
| rs1133782 | 1,24335556 | 0,97136 | 1,26101818 | 0,50858182 |
| rs3924519 | 0,1512 | 0,94176552 | 1,29054545 | 1,05370323 |
| rs4871850 | 1,32618182 | 0,74225455 | 1,30526667 | 0,99014054 |
| rs7014131 | 1,20134737 | 0,84511304 | 0,616 | 0,76418462 |
| rs7462795 | 1,12 | 1,05802667 | 1,44228 | 0,792064 |

Abbreviations: SNP ID, SNP identification; P_BH_, P values correct with Benjamini and Hochberg.

Departures from Hardy-Weinberg Equilibrium were tested using an exact test as Wigginton et al. P values were corrected for multiple testing using the Benjamini and Hochberg method. Values in bold indicates a deviation from the Hardy-Weinberg equilibrium. A hyphen indicates that the Hardy-Weinberg Equilibrium could not be calculated due to the lack of one or two genotypes.
