# Supplementary material for: TRAIL/TRAIL Receptor System and Susceptibility to Multiple Sclerosis
Source: PLoS One. 2011 Jul 21;6(7):e21766. doi: 10.1371/journal.pone.0021766 (PMC3140982; doi:10.1371/journal.pone.0021766)
Supplement: Table S3 — Abbreviations: SNP ID, SNP identification; OR = odds ratio to be replicated. (DOC) [file pone.0021766.s003.doc]

**Table S3. Power of the validation cohort to replicate significant results from the original cohort.**

| SNP ID | Disease Allele | Frequency | OR | Model | Power (%) |
| --- | --- | --- | --- | --- | --- |
| rs4894559 | A | 0.15 | 1.3 | Additive | 33.32 |
| rs4894559 | A | 0.15 | 2.37 | Recessive | 42.60 |
| rs4872077 | C | 0.43 | 1.6 | Recessive | 60.30 |
| rs11779484 | C | 0.08 | 0.71 | Dominant | 26.41 |
| rs1001793 | A | 0.52 | 0.84 | Additive | 29.00 |
| rs4460370 | T | 0.29 | 1.21 | Additive | 29.51 |
| rs4460370 | T | 0.29 | 1.56 | Recessive | 33.55 |
| rs9314261 | A | 0.19 | 0.78 | Additive | 32.75 |
| rs9314261 | A | 0.19 | 0.73 | Dominant | 38.21 |
| rs3924519 | C | 0.32 | 0.59 | Recessive | 37.60 |

Abbreviations: SNP ID, SNP identification; OR= odds ratio to be replicated.
